# Severe Congenital Neutropenia With Negative Whole-Exome Sequencing Managed With Hematopoietic Stem Cell Transplantation: A Case Report

**DOI:** 10.7759/cureus.102496

**Published:** 2026-01-28

**Authors:** Rawia F Albar, Abdulaziz A Abdulaziz, Abdulrahman H Merdad, Badr S Felemban

**Affiliations:** 1 Pediatrics, King Abdulaziz Medical City, Jeddah, SAU; 2 College of Medicine, Ministry of National Guard Health Affairs, Jeddah, SAU; 3 College of Medicine, King Abdullah International Medical Research Center, Jeddah, SAU; 4 College of Medicine, King Saud Bin Abdulaziz University for Health Sciences, Jeddah, SAU; 5 Collage of Medicine, King Saud Bin Abdulaziz University for Health Sciences, Jeddah, SAU

**Keywords:** granulocyte colony-stimulating factor (g-csf), haploidentical stem cell transplantation, kostmann syndrome, monosomy 7, severe congenital neutropenia

## Abstract

Kostmann syndrome, also known as severe congenital neutropenia, is a congenital disorder characterized by genetic mutations that prevent the progression of myeloid differentiation in the bone marrow. Most cases are associated with specific genetic mutations, including those in HAX1 and ELANE. Treatment with antibiotics and granulocyte colony-stimulating factor (G-CSF) is primarily prophylactic. We report a pediatric case of severe congenital neutropenia present since birth, with negative whole-exome sequencing (WES), complicated by multiple hospital admissions for recurrent infections and subsequent progression to myelodysplastic syndrome with excess blasts associated with monosomy 7, for which the patient ultimately underwent hematopoietic stem cell transplantation (HSCT). This case highlights that many patients with Kostmann syndrome can present with negative genetic testing and draws attention to the importance of surveillance for malignant transformation.

## Introduction

Severe congenital neutropenia, also known as Kostmann syndrome, is a rare inherited hematologic disorder characterized by a markedly decreased neutrophil count. It can be inherited through different single-gene disorders, including autosomal dominant, autosomal recessive, X-linked, and sporadic patterns. The underlying pathophysiology is characterized by an arrest of myeloid maturation in the bone marrow, resulting in markedly reduced circulating neutrophil counts [1]. Clinically, the disease is characterized by recurrent severe infections, including abscess formation, pneumonia, cellulitis, and osteomyelitis. In addition to infectious complications, specific genetic mutations are associated with distinct extra-hematologic features; for example, HAX1 mutations have been linked to neurologic involvement, whereas mutations in G6PC3 may present with cutaneous abnormalities [2]. The diagnosis is established based on a history of recurrent infections since birth alongside laboratory findings of severe neutropenia, defined as an absolute neutrophil count (ANC) below 0.5 × 10⁹/L. However, bone marrow examination remains a critical component for excluding malignant etiologies and typically demonstrates maturation arrest of the myeloid lineage [3]. Initial management consists of granulocyte colony-stimulating factor (G-CSF) therapy in patients with severe neutropenia, aiming to achieve an ANC greater than 1.0 × 10⁹/L, along with antimicrobial therapy to prevent recurrent infections [4]. Importantly, patients with severe congenital neutropenia are at increased risk of malignant transformation, with potential progression to myelodysplastic syndrome or acute myeloid leukemia.

## Case presentation

**Figure 1:**
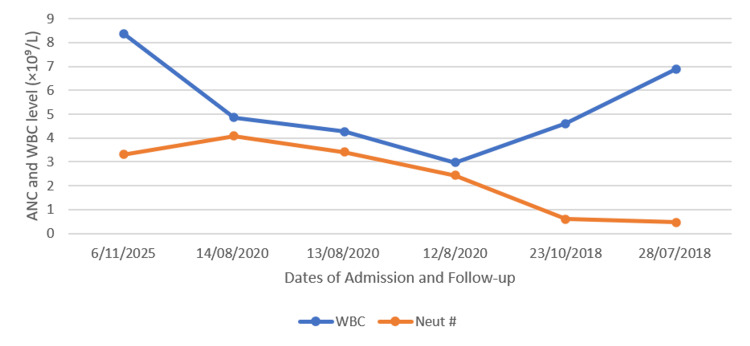
Absolute neutrophil count and WBC trends over time. Serial absolute neutrophil count (ANC) and WBC levels over the disease course. Laboratory values (×10⁹/L) are shown at major clinical milestones, including initial presentation, pre-transplant evaluation, and long-term post-stem cell transplantation follow-up, highlighting severe neutropenia prior to transplantation and sustained hematologic recovery thereafter.

A seven-year-old girl, born full term via spontaneous vaginal delivery, with no history of admission to the neonatal intensive care unit. She is a known case of severe congenital neutropenia and has a strong family history of the same condition. The patient was initially admitted in October 2018, at two months of age, with *Pseudomonas aeruginosa*-associated necrotizing fasciitis, for which she underwent surgical debridement. At presentation, laboratory investigations demonstrated severe neutropenia, with an ANC ranging from 0.4 to 0.6 × 10⁹/L and a WBC count of 7.0 × 10⁹/L (Figure 1). During the hospital course, the patient developed early osteomyelitis of the inferior pubic rami. Furthermore, an abscess was drained from the lateral aspect of the left leg, yielding pan-sensitive *Pseudomonas aeruginosa*. Hence, she was treated with intravenous ceftazidime during her hospital stay, followed by oral ciprofloxacin upon discharge. In addition, she developed a UTI caused by *Pseudomonas aeruginosa*, initially managed with vancomycin, meropenem, and clindamycin, then de-escalated to ceftazidime based on culture sensitivities. Since then, the patient has been diagnosed with severe congenital neutropenia.

Since the diagnosis, the patient has had multiple hospital admissions for different infectious complications, including febrile neutropenia, fever of unknown origin, and COVID-19 infection, all of which were managed appropriately.

The family history is notable for severe congenital neutropenia (Kostmann syndrome) and transformation to leukemia in three paternal cousins. Two of the cousins underwent HSCT; one is currently alive, while the other died. The third cousin has not yet undergone transplantation and remains on G-CSF therapy.

Genetic evaluation, including WES and targeted mutation analysis, did not identify any pathogenic abnormality. Human leukocyte antigen (HLA) typing revealed no matched related donor in the immediate or extended family. The best available match identified through the Saudi and international registries was an 8/10-matched unrelated donor.

Management

The patient was started on G-CSF at an initial dose of 5 µg/kg/day, which was then gradually up-titrated until she reached a maintenance dose of 500 µg (roughly 56 µg/kg/day) for the long term. However, in 2020, G-CSF was discontinued prior to stem cell transplantation (SCT).

In 2020, the patient was admitted for haploidentical bone marrow SCT from her mother. She underwent dental, ophthalmology, and cardiology clearance before the procedure. Additionally, a bone marrow assessment demonstrated an increase in blasts (13.5%) and significant dysmegakaryopoiesis; this finding is consistent with myelodysplastic syndrome with excess blasts (MDS-EB) and is associated with monosomy 7. The patient started conditioning therapy, which included cyclophosphamide at 50 mg/kg for two doses and busulfan at 1.2 mg/kg every six hours, in conjunction with a prophylactic loading dose of levetiracetam. Eventually, stem cell infusion was performed without complications, and the patient received a graft-versus-host disease (GVHD) prophylactic regimen during hospital admission, including cyclophosphamide, cyclosporine, and mycophenolate. Subsequently, she developed grade three cutaneous involvement and grade one ocular involvement, which were managed with systemic corticosteroids and ruxolitinib, resulting in adequate clinical control of symptoms. The patient demonstrated a favorable response to therapy, with no progression of GVHD and no evidence of chronic GVHD during follow-up. Following SCT, ANC levels improved to a range of 2.4 × 10⁹/L to 4.0 × 10⁹/L, and WBC counts to approximately 3.0 × 10⁹/L to 4.8 × 10⁹/L (Figure 1).

Follow-up

At five years post-SCT, the patient has had no further hospital admissions. In November 2025, during a recent outpatient follow-up, she was clinically stable with no active issues, had discontinued all medications, and demonstrated hematologic recovery, with an ANC of 3.31 × 10⁹/L and a WBC count of 8.38 × 10⁹/L (Figure 1).

## Discussion

Kostmann disease is a rare syndrome characterized by bone marrow failure and arrest of myeloid cells [1]. Recurrent life-threatening infections are the hallmark of the disease. In our patient, the occurrence of *Pseudomonas aeruginosa* necrotizing fasciitis in early infancy represents a severe infectious manifestation that is highly suggestive of underlying congenital neutropenia. A wide spectrum of genetic mutations has been linked to the disease, most commonly HAX1 and ELANE, while mutations in GFI1, WAS, SBDS, and G6PC3 are less common [5]. Nevertheless, up to 40% of cases are negative on genetic testing. This was observed in our patient, highlighting that a negative genetic workup does not exclude the diagnosis [2]. Although G-CSF therapy helps restore ANC to near-normal levels, the disease can transform into malignant disorders over time; thus, regular follow-up can help identify malignant transformation early [4]. In our patient, the development of MDS with excess blasts and monosomy 7 represented high-risk disease evolution, necessitating definitive intervention.

Although G-CSF therapy has demonstrated a high success rate in elevating the ANC, there are still cases in which it is not effective. While it is challenging to find a matched donor, bone marrow SCT is, in the meantime, the only curative option for the disease. However, gene therapy using CRISPR/Cas9 technology is a potential option for patients with congenital neutropenia to restore normal ANC [6]. Our patient was initially treated with G-CSF therapy for an extended period and then underwent SCT, which helped decrease the frequency of infections and hospital admissions and achieved a stable ANC.

## Conclusions

Despite improvements in genetic testing, WES is unable to identify a pathogenic variant in many patients with severe congenital neutropenia, as demonstrated in this case, suggesting the possibility of unidentified disease-associated mutations.

This case highlights the importance of continuous surveillance, alongside clinical evaluation and follow-up bone marrow examinations, for the early detection of newly acquired cytogenetic alterations, such as trisomy 21 or monosomy 7, and somatic mutations associated with leukemic transformation.
